# Optimal pathological response indicated better long-term outcome among patients with stage IB2 to IIB cervical cancer submitted to neoadjuvant chemotherapy

**DOI:** 10.1038/srep28278

**Published:** 2016-06-21

**Authors:** Kecheng Huang, Haiying Sun, Zhilan Chen, Xiong Li, ShaoShuai Wang, Xiaolin Zhao, Fangxu Tang, Yao Jia, Ting Hu, Xiaofang Du, Haoran Wang, Zhiyong Lu, Jia Huang, Juan Gui, Xiaoli Wang, Shasha Zhou, Lin Wang, Jincheng Zhang, Lili Guo, Ru Yang, Jian Shen, Qinghua Zhang, Shuang Li, Shixuan Wang

**Affiliations:** 1 Department of Obstetrics and Gynecology, Tongji Hospital, Tongji Medical College, Huazhong University of Science and Technology, Wuhan, Hubei, 430030, China; 2 Department of Obstetrics and Gynecology, Wuhan General Hospital of Guangzhou Military Command, Wuhan, Hubei, 430070, China; 3 Department of Obstetrics and Gynecology, Wuhan Central Hospital, Wuhan, Hubei, 430014, China; 4 Department of Orthopedics, Tai-He Hospital, Hubei University of Medicine, Shiyan, Hubei, 442000, China; 5 Department of Internal Medicine, Luohe Renmin Hospital, the First Affiliated Hospital of Luohe Medical College, Luohe, Henan, 462000, China; 6 Tai-He Hospital, Hubei University of Medicine, Shiyan, Hubei, 442000, China; 7 Department of Obstetrics and Gynecology, Renmin Hospital, Wuhan University, Wuhan, Hubei, 430000, China; 8 Xinhua Hospital, Shanghai Jiao Tong University School of Medicine, Shanghai, 200092, China; 9 Henan Cancer Hospital, Zhengzhou, Henan, 450008, China

## Abstract

The role of pathological response in long-term outcome is still unclear in cervical cancer patients treated with neoadjuvant chemotherapy (NACT) in China. This study aimed to investigate the effect of optimal pathologic response (OPR) on survival in the patients treated with NACT and radical hysterectomy. First, 853 patients with stage IB2-IIB cervical cancer were included in a retrospective analysis; a Cox proportional hazards model was used to investigate the relationship between pathological response and disease-free survival (DFS). In the retrospective database, 64 (7.5%) patients were found to have achieved an OPR (residual disease <3 mm stromal invasion); in the multivariate Cox model, the risk of death was much greater in the non-OPR group than in the OPR group (HR, 2.61; 95%CI, 1.06 to 6.45; *P* = 0.037). Next, the role of OPR was also evaluated in a prospective cohort of 603 patients with cervical cancer. In the prospective cohort, 56 (9.3%) patients were found to have achieved an OPR; the log-rank tests showed that the risk of recurrence was higher in the non-OPR patients than in the OPR group (*P* = 0.05). After combined analysis, OPR in cervical cancer was found to be an independent prognostic factor for DFS.

Cervical cancer is the second most commonly diagnosed malignancy and the third leading cause of cancer deaths in less developed areas. It has been estimated that there were 527,600 new cervical cancer patients and 265,700 deaths around the world in 2012^1^. In China, cervical cancer had a cancer prevalence estimates for 5 years with 313,700 cases in 2011^2^. Concomitant chemo-radiotherapy (CCRT) is the gold standard therapy for locally advanced cervical cancer (LACC). However, neoadjuvant chemotherapy (NACT) has emerged as a promising step forward in the management of cervical cancer^3^,^4^,^5^. As precision radiotherapy units are particularly rare in developing areas, such as in rural areas of China, doctors have to resort to neo-adjuvant chemotherapy to shrink tumours for surgical performance^6^,^7^,^8^,^9^,^10^,^11^. Quite a few studies have also investigated this innovation, including randomized clinical trials and cohort and case-control studies across the world^6^,^7^,^8^,^9^,^12^,^13^,^14^,^15^,^16^,^17^,^18^. Furthermore, NACT provides an opportunity to optimize therapy, especially for fertility-preserving therapy^19^,^20^. This favourable result of NACT may lead to a new era of LACC treatment.

In addition, NACT also helps clinicians assess tumour response to a particular chemotherapeutic regimen^9^,^10^,^13^,^21^,^22^,^23^. Previous studies in western areas have concluded that an optimal pathological response (OPR) may be a prognostic factor for survival in cervical cancer^12^,^24^. However, few studies have examined the impact of OPR on survival in Chinese patients, and no studies have performed such an assessment with a sufficiently large sample size to draw a definitive conclusion.

Therefore, we designed a retrospective study to assess whether pathological response affected survival in Chinese patients with International Federation of Gynecology and Obstetrics (FIGO) stage IB2-IIB cervical cancer treated with neo-adjuvant chemotherapy and radical hysterectomy; additionally, we validated the effect of pathological response in a prospective cohort.

## Results

### Patient characteristics

In the retrospective analysis, we included 853 patients with stage IB2-IIB cervical cancer receiving neo-adjuvant platinum-based chemotherapy and radical hysterectomy (Table 1). The median age of the patients at the time of study entry was 44 (range 39–50) years. Of the 853 patients, 64 (7.5%) achieved an OPR, and the other 789 did not. In the prospective cohort, 603 patients were included, all of whom underwent neo-adjuvant platinum-based chemotherapy and radical hysterectomy; the details are shown in Table 1.

### Univariate Cox proportional hazards model for DFS

A Cox proportional hazards model was used to investigate whether clinical variables and pathological response affected the DFS. In the univariate Cox analysis of the retrospective study, pathological response achieved statistical significance for the DFS (HR 11.05, *P* = 0.02 in Table 2, respectively). For the prospective cohort study, the pathological response also achieved statistical significance for the DFS (HR 3.65, *P* = 0.07 in Table 3).

### Multivariate Cox proportional hazards model for DFS

In the multivariate analysis of the retrospective cohort, we observed that pathological response was associated with the DFS rate (HR 2.61, *P* = 0.037 in Table 4). In the prospective cohort study, the pathological response also achieved higher DFS result but without statistical significance (HR 4.03, *P* = 0.053 in Table 5).

### Log-rank test for DFS in the retrospective study and in the prospective cohort

**Figure 1:**
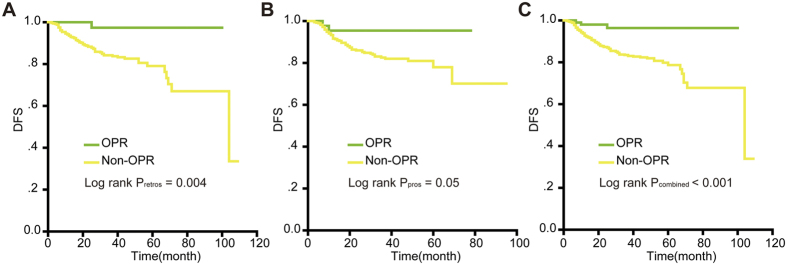
Kaplan-Meier survival estimates for OPR and Non-OPR patients with cervical cancer from the retrospective study, the prospective cohort and the combination of the two studies. Disease-free survival (DFS) curves of evaluated patients in the retrospective study (**A**), the prospective cohort (**B**) and the combined results (**C**). Log-rank test used to calculate *P* values. *P* < 0.05 was considered to be statistically significant.

DFS rates were compared using the Kaplan-Meier method for the OPR and non-OPR groups; the *P* values for DFS were 0.004 in the retrospective study (Fig. 1A). Later, information from the cervical cancer patients in the prospective cohort was used to assess the role of OPR in DFS (*P* *=* 0.05, Fig. 1B). Figure 1C showed when the retrospective study and the prospective study were combined, OPR patients achieved a significantly higher survival rate than non-OPR patients (*P* < 0.001 for DFS).

### Joint analysis of the retrospective study and the prospective cohort

**Figure 2:**
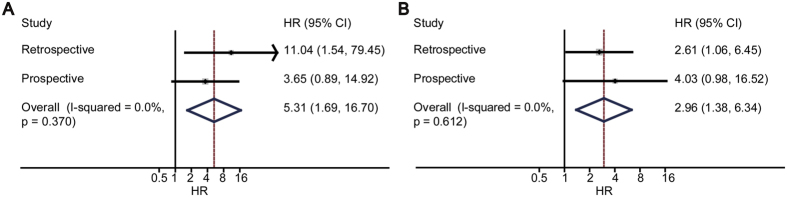
The combined results of Non-OPR and cancer-recurrence risk. For univariate Cox regression, the summary relative risk was 5.31 (95% CI, 1.69 to 16.70) and test of heterogeneity I^2^ = 0% (*P* = 0.37) (**A**); for multivariate Cox regression, the summary relative risk was 2.96 (95% CI, 1.38 to 6.34) and test of heterogeneity I^2^ = 0% (*P* = 0.61) (**B**). The combined analysis showed that Non-OPR was statistically associated with recurrence.

The results from the retrospective study and the prospective study were combined together according to the method illustrated in the previous study^25^. In univariate Cox analysis, HR got a value of 5.31 (95% CI, 1.69 to 16.70) (Fig. 2A). In multivariate Cox analysis, HR got a value of 2.96 (95% CI, 1.38 to 6.34) (Fig. 2B).

## Discussion

We combined both a retrospective study and a prospective cohort to assess the value of pathological response; this study comprehensively examined the pathological response to neo-adjuvant chemotherapy and evaluated its value in predicting long-term disease-free survival.

When analysed using a univariate Cox proportional hazards model, our retrospective data indicated that one of the most important predictors of long-term prognosis was the invasion depth of residual cancer cells upon the completion of treatment. The condition of ≤3 mm of invasive tumour (OPR) in the cervix was demonstrated to be related to improved survival. Patients who achieved OPR exhibited excellent survival, and patients without OPR after neo-adjuvant chemotherapy demonstrated significantly shorter DFS times. This effect resulted in an increase in the 3-year DFS rate. From the multivariate analysis, OPR was also found to serve as an independent prognostic factor with a high HR. The HR was similar to that of a previous study^12^. The combined results of the Kaplan-Meier log-rank test indicated that the OPR group demonstrated significantly improved DFS rate. For further assessment of the pathological response in cervical cancer, the results of a clinical trial should be sufficiently discussed^12^. The clinical trial also proved that OPR was an independent prognostic factor of survival^12^. The trial demonstrated a significant OS benefit at 5 years for patients experiencing an OPR versus patients who did not experience an OPR. The average death rates were significantly higher in the group that did not achieve an OPR than in the group that did achieve an OPR. A previous study also suggested that obtaining an OPR was a beneficial prognostic factor of long-term survival^24^,^26^. Other research studies, both prospective and retrospective, have also demonstrated complete or optimal partial pathologic response after neo-adjuvant systemic treatment to be associated with a higher chance of cervical cancer survival^27^,^28^. The OPR rate in our study was relatively lower than those of previous studies; this was mainly attributed to the fact that the majority of patients only received one cycle, and thus these patients may not have had the chance to respond.

A growing number of studies have also evaluated the relationship between pathological response and other malignant disease outcomes. Furthermore, pathological response has been increasingly adopted as a measure of activity for NACT and also as a prognostic factor for survival, such as in breast cancer studies. The researchers of a triple-negative breast cancer study observed that patients with a pathological complete response (PCR) demonstrated excellent survival compared with patients without a PCR^29^. Other researchers have noted that PCR could be used as an early surrogate marker for long-term survival in invasive breast cancer after neo-adjuvant chemotherapy^30^. Moreover, a group of researchers performed a meta-analysis of PCR associated with colon cancer outcomes and reported that patients with a PCR after chemoradiation demonstrated better long-term outcomes than those without a PCR in colon cancer^31^. Numerous results in the literature thus suggest that pathological response can affect the outcome of survival^30^,^31^,^32^. Researchers have also suggested that a PCR might be indicative of a prognostically favourable biological profile with fewer propensities for local or distant recurrence and improved survival. Other researchers have observed that a complete or optimal pathological response is associated with good survival outcomes^27^,^33^.

Therefore, OPR may serve as a useful prognostic indicator for cervical cancer patients who receive neo-adjuvant chemotherapy. The aim of systemic chemotherapy is to eliminate the primary tumour and to eradicate residual occult distant metastasis to ultimately improve DFS and OS. Theoretically, if an OPR is reflective of chemotherapy sensitivity in occult distant sites, patients who exhibit an OPR in their primary tumour would demonstrate the highest DFS rate. This relationship has been demonstrated in our study, as OPR was associated with a better pathological outcome.

The study included an adequate sample size for the main outcomes. However, there were some limitations. First, our study did not integrate biologic makers associated with cancer progression and survival; we considered only certain clinical factors. No biological markers were included on the gene, mRNA or protein level. Second, new surgeries, such as radical trachelectomy, should also be carefully investigated in our medical centre. To address these problems, biomarkers should be added to our studies in the future, and at the same time, new updates should be made to our database.

As is widely known, NACT may lead to excellent survival in a particular group of patients, such as the group of patients with OPR. For patients with obvious node positive disease, NACT may place them at a high risk for delaying optimal treatment. The standard treatment of LACC is chemo-radiation (CCRT), and CCRT has also shown superior outcomes in long-term survival. Therefore, doctors should perform thorough pre-treatment evaluations to identify the most suitable patients, such as OPR patients. As for the patients who are less likely to achieve OPR or clinical response after NACT, CCRT may be the appropriate therapy to pursue without exposing them to delayed therapy, such as NACT. To identify OPR patients, clinicians in our department are trying to develop a predictive model that is able to identify the patients who are most likely to achieve OPR, clinical response or non-response.

Last but not least, clinicians and scientists should pay close attention to patients who do not achieve OPR after NACT. These patients are less sensitive to neo-adjuvant chemotherapy, and adjuvant post-surgery treatment such as CCRT should be particularly considered. The clinical-pathologic risk factors such as positive lymph nodes, low-grade differentiation, and parametrial infiltration, as well as GOG score should be reviewed together to make a decision on adjuvant post-surgery treatment^34^.

With the aim of identifying the pathological predictors of long-term survival and postoperative management effects, we conducted a research study and demonstrated that OPR is a predictor of a good prognosis. The results revealed that OPR after neoadjuvant chemotherapy can improve long-term outcomes, with fewer possibilities of recurrence, and can increase long-term survival compared with patients without an OPR. To obtain further evidence, additional prospective studies in different nations are necessary, and a model to predict OPR or clinical response is also necessary.

## Methods

### Study Design

First, medical records were retrospectively reviewed from a database on cervical cancer, which consisted of clinical data from 853 patients. Then, medical information was reviewed from a prospective cohort, which consisted of recently updated clinical data (http://clinicaltrials.gov; NCT01628757); 603 patients fulfilled the inclusion criteria after exclusion. The following data were retrieved from the database, patient files, and pathology reports: age at diagnosis, year of treatment, stage of disease, cell type, grade of differentiation, tumour size, lymph node involvement, parametrial involvement, depth of tumour invasion, lymphovascular space invasion (LVSI), surgical margin status, adjuvant treatment, and follow-up status.

This study followed the declaration of Helsinki and was conducted in accordance with the approved guidelines. All experimental protocols were approved by the ethical committee of Tongji Medical College at Huazhong University of Science and Technology. All eligible patients provided written informed consent before entering this study.

### Inclusion criteria

We enrolled patients based on the following criteria: 1. patients with International Federation of Gynecology and Obstetrics (FIGO) stage IB2-IIB cervical cancer; 2. patients less than 70 years old; 3. patients treated with NACT followed by radical hysterectomy; and 4. patients not receiving primary radiotherapy, concurrent chemo-radiotherapy or preoperative radiotherapy and those without complicating disease including renal failure and hepatic failure or prior malignant disease. Surgery was performed within 4 weeks of completing the last course of chemotherapy. All patients received radical hysterectomy with pelvic lymphadenectomy, and para-aortic lymphadenectomy was performed in patients with suspicious para-aortic lymph node metastasis.

### Exclusion criteria

The exclusion criteria included a Karnofsky Performance Status <70, age less than 18 years old, previous history of cancer, or previous treatment of cancer (i.e., surgery, chemotherapy or radiotherapy). Patients with active infectious disease or other medical complications including hepatic failure and renal failure and women who lacked information on clinical risk factors were also excluded from this study.

### Pretreatment and post-treatment evaluation

The diagnosis was confirmed by pathological experts for each patient according to cervical biopsy and staged as IB to IIB by clinicians according to the International Federation of Gynaecology and Obstetrics (FIGO). Tumour status was checked clinically, and EKG was performed when each treatment began. An ultrasound of the tumour and pelvic condition was scheduled after each cycle to control for progressive disease in all patients. If the tumours were considered operable, radical surgery was performed within 4 weeks of completion of the last scheduled chemotherapy cycle. Otherwise, the patients received concurrent chemoradiotherapy.

### Neoadjuvant chemotherapy

The standard treatment for NACT is a platinum-based regimen, which was used in our study. NACT was administered in 1–2 courses, depending on the patient’s tolerance and response, and a small number of patients received an additional 1–2 cycles.

### Pathological response

The pathological response was retrospectively assessed as in previous studies^3^,^12^,^24^,^26^; PCR was defined as the complete disappearance of the tumour from the cervix and negative nodes; PR1, partial response one, was defined as residual disease with less than 3 mm stromal invasion, including *in situ* carcinoma with or without lymphatic metastasis; and PR2, partial response two, was defined as persistent residual disease with more than 3 mm stromal invasion in the surgical specimen. Studies chose 3 mm as the lowest limit of OPR because it represents the maximal extension of FIGO stage IA1 cervical cancer^12^,^32^. OPR was defined as PCR+PR1. The histopathological diagnosis was confirmed by two pathologists for each patient in our study. In addition, the assessment of OPR was based on the histopathological diagnosis. In our study, we investigated the influence of OPR on survival.

### Follow-up study

The follow-up of patients was designed to be conducted every 3 months in the first year and every 6 months in the next four years after surgery. According to our database, for a small proportion of patients, follow-up was not performed due to loss of contact, and the data from these individuals were excluded from the survival analysis. The DFS rate was calculated from the day of diagnosis until the date of first relapse or death (regardless of any cause)^35^.

### Statistical analysis

The primary goal for this analysis was the relationship between response and DFS. When testing at the 0.05 level (two-sided test), the combined sample size (both the retrospective study and prospective cohort) would provide a statistical power more than 90% to detect the statistical difference for DFS with the hypothesis (HR = 2.5).

Log-rank tests were used for the DFS comparisons. A Cox proportional hazards model was used for multiple regression analysis to verify whether the clinical variables and the pathological response variable predicted DFS. In the multivariate models, variables were automatically retained by the computer if their associated multivariate *P* values were less than 0.05 or if they were necessary for the model. The median follow-up time was calculated as the median observation time among all patients. IBM SPSS 20.0 software was used to perform the statistical analyses. All reported *P*-values were two-sided, and we considered *P* < 0.05 to be significant.

## Additional Information

**How to cite this article**: Huang, K. *et al*. Optimal pathological response indicated better long-term outcome among patients with stage IB2 to IIB cervical cancer submitted to neoadjuvant chemotherapy. *Sci. Rep.* **6**, 28278; doi: 10.1038/srep28278 (2016).

## Figures and Tables

**Table 1:** Clinical characteristics for all patients.

**Characteristics**	**Retrospective study(n = 853)**		**Prospective cohort study(n = 603)**	
	**No.**	**%**	**No.**	**%**
Age(25th–75th percentiles) (year)				
Median	44		45	
Range	39–50		41–51	
Age (year)				
20–30	36	4.2	18	3.0
30–40	218	25.6	126	20.9
40–50	403	47.2	306	50.6
50–60	165	19.3	131	21.6
60–70	31	3.6	22	3.6
Tumor size(25th–75th percentiles) (cm)				
Median	4.0		4.0	
Range	3.5–5.0		3.0–5.0	
Tumor grade				
G1	58	6.8	42	7.0
G2	354	41.5	242	40.1
G3	240	28.1	185	30.7
Undetermined	201	23.6	134	22.2
FIGO stage				
IB2	220	25.8	134	22.2
IIA	265	31.1	129	21.4
IIB	368	43.1	340	56.4
Cell type				
Squamous	756	88.6	533	88.4
Non-squamous	91	10.7	60	10.0
Unknown	6	0.7	10	1.6
Pathological response				
OPR	64	7.5	56	9.3
non-OPR	789	92.5	542	89.9
Unknown			5	0.8

FIGO, International Federation of Gynecology and Obstetrics.

**Table 2:** Univariate Cox regression for DFS in the retrospective study.

**Variables**		**HR**	**95% CI**	**P**
Pathological response	OPR VS Non-OPR	11.05	1.54 to 79.45	0.02
Age	>44 VS ≤44 years	1.61	1.05 to 2.48	0.03
Stage	IIA VS IB2	2.18	1.13 to 4.20	0.02
	IIB2 VS IB2	2.44	1.33 to 4.48	0.004
Tumor size	>4 cm VS ≤4 cm	1.37	0.86 to 2.19	0.19
Grade	G2 VS G1	2.16	0.67 to 7.01	0.20
	G3 VS G1	3.38	1.06 to 10.81	0.04
	Undetermined VS G1	2.05	0.60 to 6.97	0.25
Cell type	Squamous VS non-squamous	2.24	1.32 to 3.82	0.003
LVSI	Positive VS negative	1.40	0.75 to 2.61	0.29
Parametrial infiltration	Positive VS negative	2.61	1.53 to 4.44	<0.001
Vaginal surgical margin	Positive VS negative	1.91	0.83 to 4.41	0.13
Lymph node metastasis	Positive VS negative	3.68	2.21 to 6.12	<0.001

LVSI, Lymph vascular space invasion. DFS, disease free survival.

**Table 3:** Univariate Cox regression for DFS in the prospective study.

**Variables**		**HR**	**95% CI**	**P**
Pathological response	OPR VS Non-OPR	3.65	0.89 to 14.92	0.07
Age	>44 VS ≤44 years	2.18	1.30 to 3.67	0.003
Stage	IIA VS IB2	1.61	0.64 to 4.09	0.31
	IIB2 VS IB2	2.56	1.20 to 5.43	0.01
Tumor size	>4 cm VS ≤4 cm	0.93	0.56 to 11.57	0.23
Grade	G2 VS G1	1.30	0.39 to 4.36	0.69
	G3 VS G1	1.94	0.58 to 6.55	0.28
	Undetermined VS G1	2.40	0.70 to 8.20	0.16
Cell type	Squamous VS non-squamous	1.45	0.68 to 3.12	0.34
LVSI	Positive VS negative	2.48	0.92 to 6.68	0.07
Parametrial infiltration	Positive VS negative	3.32	1.20 to 9.16	0.02
Vaginal surgical margin	Positive VS negative	4.04	1.71 to 9.54	0.001
Lymph node metastasis	Positive VS negative	2.62	1.44 to 4.78	0.002

LVSI, Lymph vascular space invasion; DFS, disease free survival.

**Table 4:** Multivariate Cox regression for DFS in the retrospective study.

**Variables**	**Retrospective study**			
	**B**	**HR(95% CI)**	**P**	
Pathological response				
OPR		1		
Non-OPR	0.96	2.61(1.06,6.45)	0.037	
FIGO stage				
IB2		1		
IIA	0.60	1.81(1.19,2.78)	0.006	
IIB	0.44	1.55(1.03,2.34)	0.038	
Grade				
G1		1		
G2	0.50	1.61(0.77,3.38)	0.20	
G3	1.11	3.05(1.46,6.34)	0.003	
Undetermined	0.22	1.24(0.56,2.75)	0.60	
Cell type				
Squamous		1		
Non-squamous	0.56	1.76(1.18,2.61)	0.005	
Lymph node metastasis				
Negative		1		
Positive	0.58	1.78(1.30,2.42)	<0.001	

FIGO, International Federation of Gynecology and Obstetrics; DFS, disease free survival.

**Table 5:** Multivariate Cox regression for DFS in the prospective study.

**Variables**	**Prospective cohort study**			
	**B**	**HR(95% CI)**	**P**	
Pathological response				
OPR		1		
Non-OPR	1.39	4.03(0.98,16.52)	0.053	
Age				
≤44 years		1		
>44	0.89	2.43(1.44,4.13)	0.001	
Lymph node metastasis				
Negative		1		
Positive	0.43	1.54(1.01,2.36)	0.045	

DFS, disease free survival.
